# Frizzled-10 Extracellular Vesicles Plasma Concentration Is Associated with Tumoral Progression in Patients with Colorectal and Gastric Cancer

**DOI:** 10.1155/2019/2715968

**Published:** 2019-06-02

**Authors:** Maria Principia Scavo, Antonio Cigliano, Nicoletta Depalo, Elisabetta Fanizza, Maria Grazia Bianco, Nunzio Denora, Valentino Laquintana, Maria Lucia Curri, Dionigi Lorusso, Claudio Lotesoriere, Alba Panarese, Gianluigi Giannelli

**Affiliations:** ^1^Personalized Medicine Laboratory, National Institute of Gastroenterology “S. De Bellis” Research Hospital, Via Turi 27, Castellana Grotte, Bari, Italy; ^2^Institute for Chemical-Physical Processes (IPCF)-CNR, SS Bari, Via Orabona 4, 70125 Bari, Italy; ^3^Università degli Studi di Bari Aldo Moro, Dipartimento di Chimica, Via Orabona 4, 70125 Bari, Italy; ^4^Università degli Studi di Bari Aldo Moro, Dipartimento di Farmacia, Scienze del Farmaco, Via Orabona 4, 70125 Bari, Italy; ^5^Surgical Gastroenterology Unit, National Institute of Gastroenterology “S. De Bellis” Research Hospital, Via Turi 27, Castellana Grotte, Bari, Italy; ^6^Oncology Unit, National Institute of Gastroenterology “S. De Bellis” Research Hospital, Via Turi 27, Castellana Grotte, Bari, Italy; ^7^Endoscopy Unit, National Institute of Gastroenterology “S. De Bellis” Research Hospital, Via Turi 27, Castellana Grotte, Bari, Italy; ^8^Scientific Direction, National Institute of Gastroenterology “S. De Bellis” Research Hospital, Via Turi 27, Castellana Grotte, Bari, Italy

## Abstract

Extracellular vesicles (EVs) are involved in intercellular communication during the carcinogenesis. Our attention has been focused on small EVs (sEVs) protein content in colorectal and gastric cancer (CRC and GC). Frizzled (FZD) proteins, a family of receptors comprised in the Wnt signaling pathway, play an important role in the carcinogenesis of CRC and GC. Here, the expression of a specific FZD protein, namely, FZD-10, was investigated in the sEVs extracted from plasma of patients affected by CRC and GC as involved in canonical and noncanonical Wnt signaling in cancer stem cells with a subsequent modification of cellular heterogeneity, omics reprogramming, and tumor plasticity. The expression of FZD-10 protein in the sEVs extracted from plasma of patients affected by CRC and GC and sEVs from plasma of healthy subjects was evaluated against the level of protein Hsp70, established as EVs specific markers along with CD63 and ALIX proteins. The FZD-10 extract from sEVs isolated from plasma of the controls and the CRC or GC subjects indicated that its expression in oncological patients was higher than in the control group, while, at the end of the treatment, it reached values comparable with the average level of controls. Furthermore, the level of FZD-10 in the whole plasma was found comparable with its level in the sEVs extract. The level of FZD-10 in the sEVs represents a potential reliable biomarker with a valuable prognostic function for the diagnosis of CRC and GC and for monitoring the treatment response.

## 1. Introduction

Colorectal (CRC) and gastric (GC) cancer are the most common causes of cancer-related death worldwide, in both sexes. The development of novel and reliable screening methods for their early detection is strictly required to reduce the mortality rate by efficacious prevention and treatment before the cancer progression into advanced stages. From this perspective, researchers are attempting numerous efforts to identify new specific cancer-related biomarkers in biological fluids or their components. Extracellular vesicles (EVs) are released in extracellular space, through the cytoplasm membranes, by all types of cells, in healthy and pathological conditions [1], and can be isolated starting from different biological fluids, such as blood, saliva, cerebrospinal or amniotic fluid, and urine [2]. EVs are vesicles composed of enclosed lipid bilayer cell membranes ranging from 30 nm to 2,000 nm in diameter, which can be generally categorized into three main classes, namely, apoptotic bodies (0.5–5 *μ*m), microvesicles (50 nm–2 *μ*m), and exosomes (30–120 nm).

In the last decade different reports have shown that cancer cells secrete EVs that contain molecules, such as miRNAs, mRNA, noncoding RNA, DNA fragments, and proteins, that can be transferred to recipient cells and/or* vice versa* to induce new biological processes, in particular, therapeutic resistance, angiogenesis, formation of metastasis, (re)programming remodeling, and cancer cell plasticity modification [3–5]. Several studies have also proved the great potential of human fluid derived EVs profile, in terms of dynamic changes in content of specific EVs delivered protein related to cancer onset, as diagnostic biomarker for early cancer detection [6].

While the role of nucleic acids in EVs is well documented, the function of EVs proteins still remains not fully elucidated [7]. Although EVs are characterized by a complex, heterogeneous, and source cell type-dependent composition, specific proteins are always highly retained in EVs, regardless of the cell types that secrete them, thus resulting in EV protein markers commonly used for their characterization. These specific proteins include endosomal proteins such ALIX, TSG101, clathrin, and ubiquitin or transmembrane proteins such as integrins and tetraspanins (namely, CD9, CD63, and CD81), as well as heat shock proteins (namely, HSC70, HSP60, HSP70, and HSP90) [8]. Interestingly, different families of proteins delivered as protein cargo in purified cancer-derived EVs have been demonstrated to be involved in tumor progression, in cancer cells remodeling and metastasis development in different types of cancers and, in some cases, also identified as diagnostic cancer markers [9–13]. In particular, for GC, experimental data have suggested the involvement of the exosome CD97 protein in the promotion of GC proliferation and metastasization through exosome-mediated MAPK signaling pathway [10]. Indeed, a critical role in the development of peritoneal metastasis in GC has been ascribed to cancer-derived exosomes, through an increased expression of adhesion molecules in mesothelial cells, such as FN1 and LAMC1 proteins [14]. H. Fu et al. have suggested the role of exosomal TRIM3 protein as novel diagnostic biomarker and therapeutic target for GC [15]. In the case of CRC, some of the proteins included in PTEN/Akt pathway and present in EVs can induce also the chemotherapy resistance, with a significant PTEN downregulation and Akt phosphorylation [16]. Recently, it has been proved that EVs are able to transfer insoluble Wnt proteins between diverse cell types, thus highlighting a key roles of EVs in regulating the Wnt/*β*-catenin signaling pathway that is implicated in tumor metastasis and cancer development [17–20]. In particular, Y. B. Hu et al. have documented the ability of exosome Wnt derived from fibroblasts to induce chemoresistance in CRC, thus suggesting that the therapeutic response could be enhanced by interfering with Wnt signaling [19–21] Furthermore, Y. Tian et al. have proved the presence of a significantly elevated level of CD147-positive EVs in CRC patients compared to healthy controls, thus indicating this protein as promising marker for CRC diagnosis [22].

In this work, the attention was focused on the occurrence of Frizzled-10 (FZD-10) in EVs isolated from plasma of patients affected by sporadic GC and CRC, at different stages, with different etiology and progression, as well as at different steps of disease managements. The FZDs are a family of transmembrane receptors which play pivotal roles in Wnt pathways. Ten members (FZD-1–FZD-10) of FZD family have been identified in humans. A number of studies have demonstrated the roles of FZD family members in malignant progression of various human cancers [23]. In particular, the FZD-10 has proven to be involved in tumor development, and cancer cell remodeling with Wnt cascade, as a strong correlation between staging and protein localization in the tumors cells, has been recently highlighted not only in CRC, GC, and melanoma, but also in synovial carcinoma [23, 24].

FZD-10 is a receptor that, along with other coreceptors proteins (LRP-5 and LRP-6), is a constitutive part of Wnt receptor complex. In normal conditions, FZD-10 is involved in organogenesis during embryonic development, cell migration, neural patterning, and cell polarity [25]. After the binding of Wnt to the receptor complex, the FZD-10 interaction with a cytoplasmic phosphoprotein Dsh/Dvl [26] allows the complex to enter in the nucleus to complete the signal transduction. An increase in the FZD-10 cytoplasmic expression during the carcinogenesis has been observed and reported. In the normal tissue, the FZD-10 expression is very low, although data support the well-established relevance of this protein during the development of the vascular system and of the brain vascular endothelial cells in central nervous system angiogenesis [27].

Evidence of a possible tumor biological role of the FZD-10 includes the upregulation of FZD-10 mRNA in several types of human cells during the carcinogenesis through activation of the *β*-catenin-TCF signaling pathway, in presence of Wnt [28]. The hyperactivation of some gene transcription through Wnt pathway, like *β*-catenin-T cell factor (TCF)/ lymphoid enhancer factor (LEF)-regulated gene transcription, is a peculiar sign of CRC development. *β*-catenin is also a key regulator of cell-cell adhesion, by binding the E-cadherin transmembrane adhesion receptor that is involved in cell-cell interaction bridges to the actin cytoskeleton [29].

Here, the EVs isolated from plasma of healthy and oncological patients were fully characterized in terms of size, morphology, and surface charge by means of TEM, SEM, DLS, and *ζ*-potential measurements. The mean size values recorded for the EVs isolated from plasma of healthy and oncological patients pointed out that the applied purification protocol allows isolating the EVs fraction known as “small EVs” (sEVs), having size in the range between 100 and 200 nm, according to established nomenclature reported in the update of MISEV2014s guidelines. Subsequently, the expression level of FZD-10 in sEVs was selectively evaluated, by semiquantitative densitometric analysis, against the level of Hsp70 protein, established as EV specific protein marker along with CD63 and ALIX proteins [30].

Remarkably, for the first time, here, FZD-10 protein was found to be specifically localized in the sEVs from plasma of both oncological and healthy subjects, and not just in the canonical localization.

The results of Western blotting analysis performed on sEVs isolated from plasma of healthy donors and CRC or GC patients indicated that, in all the investigated cases, the FZD-10 level of expression in the oncological patients was higher than that recorded for the healthy control group, thus representing a clear indication of the pathological condition. Interestingly, the protein level in the sEVs from patients at the end of the treatment appears to reach values comparable with the average level recorded for the healthy donors.

Furthermore, the expression level of FZD-10 in the sEVs isolated from the plasma was estimated prior to any isolation procedure, in order to evaluate whether FZD-10 is distinctively carried by the EVs or also present in the EVs-depleted plasma.

Remarkably, the expression level of FZD-10 detected in the sEVs was found comparable with the corresponding protein level detected in the whole plasma from the same subject. This evidence proved that FZD-10 measured in the sEVs represents the actual total level of the protein in the plasma and, hence, is distinctively contained in the sEVs.

The overall results indicated that evaluation of the FZD-10 level in the sEVs could represent a reliable potential biomarker with a valuable prognostic role in the early diagnosis of CRC and GC and in monitoring the treatment response.

## 2. Materials and Methods

### 2.1. Patients and Plasma Collection

In total 30 patients were enrolled, 11 females and 19 males; each patient complying with the inclusion criteria (Table 1) signed the informed consent. In particular, 22 CRC patients (M/F:14/8, age 70.42 ± 8.7), 8 patients affected by GC (M/F:5/3, age 70.33 ± 6.65) (Table 1), and 8 healthy donors were enrolled (M/F:10/2, age 45.2 + 3.6). Patients with sporadic adenocarcinoma in colon and stomach, more than 50 years old, and women in menopause for at least 2 years were enrolled. Nonsteroidal anti-inflammatory drugs were not assumed by any patient at the time of diagnosis.

### 2.2. Extracellular Vesicles Isolation

Plasma specimens from all the subjects were processed in order to perform sEVs extraction, by following the protocol reported in [31].

Briefly, venous sampled blood specimens, from the subjects, either oncological patients or healthy volunteers, were kept at room temperature for 30 minutes, then they were centrifuged at 4°C for 10 minutes at 1500 g in ethylenediaminetetraacetic acid (EDTA).

The supernatant fluid (plasma) was transferred to a clean tube, centrifuged again at 1,800 g for 10 minutes at 4°C, and then carefully transferred into suitably labeled screw-cap cryovial. Typically, 5 mL of whole blood yielded about 1.5 mL of plasma that was then divided into aliquots of 500 *μ*L and frozen at −80°C when not processed immediately. The plasma specimens were thawed and centrifuged at 3000 g for 15 minutes at 4°C and the supernatant was transferred into a clean tube for another centrifugation cycle at 3800 g for 15 minutes at 4°C. Subsequently an ultracentrifugation (UC) cycle was performed by using a BECKMAN, L-60 Ultracentrifuge, at 75000 g for 1 hour at 4°C, then the supernatant was transferred into another clean ultracentrifuge tube, and a second centrifugation cycle was performed at 100000 g for 1 hour and 30 minutes.

The supernatant was then discarded and the pellet formed of sEVs was collected and diluted in 200 *μ*L of ultrapure water. For each sample, 50 *μ*L of sEVs suspension was immediately processed for DLS, TEM, and SEM characterization, while the left sample was stored at −80°C until the protein extraction was carried out.

### 2.3. Protein Extraction and Quantification from Plasma and Small Extracellular Vesicles

After isolation, all sEVs samples obtained were homogenized by using 1X radio immunoprecipitation buffer (RIPA; Cell Signaling Technology, Danvers, MA, US) containing protease inhibitor (Amresco, Solon, OH, US), and protein content in the homogenates was measured by means of Bradford kit assay (Bio-Rad Hercules, CA, US).

The same protocol was applied to extract and determine the total protein content in the whole plasma. The same protein amount from each sample (20 *μ*g) was mixed with reducing Laemmli buffer, loaded on 4–15% Tris-glycine sodium dodecyl sulfate-polyacrylamide gels (Bio-Rad, Hercules, CA, US), and electrophoresed. The proteins were then blotted to nitrocellulose membranes (Bio-Rad, Hercules, CA, US) using Trans-Blot System (Bio-Rad, Hercules, CA, US). The blotted membranes were treated with 5% nonfat milk (Bio-Rad, Hercules, CA, US) in Tris-buffered saline supplemented with 0.05% Tween-20 (TBS-T) for 2 h, to block aspecific sites and then incubated with primary antibodies, namely, anti-CD63 [1:1000; Abcam, Cambridge, UK], anti-FZD-10 [1:400; Abcam, Cambridge, UK,], anti-ALIX [1:1000 Abcam, Cambridge, UK], anti-Hsp70 [1:1000 Abcam, Cambridge, UK], and anti-GAPDH [1:5000 Santa Cruz, California, US] over night at 4°C. After 3 washing cycles in TBS-T, the membranes were incubated with corresponding HRP-conjugated secondary antibodies for 1 h at room temperature and subsequently washed in TBS-T. The chemiluminescence signals from proteins were imaged after incubation by using an enhanced chemiluminescence kit (Bio-Rad, Hercules, CA, US) by ChemiDoc XRS+ (Bio-Rad, Hercules, CA, US). The images were analyzed by using Image Lab 5.2.1 software.

### 2.4. Statistical Analysis

The Sigma Stat 3.1 software was used for statistical analysis. Statistical significance between two groups was assessed using the Student's t-test (unpaired), and multiple comparisons were assessed using one-way analysis of variance. When the hypothesis of the mean equality among groups was rejected by the one-way analysis of variance, the Kruskal-Wallis test was applied.

### 2.5. Transmission Electronic Microscopy (TEM) Investigation

The isolated EVs were morphologically characterized by TEM performed by using a Jeol JEM-1011 microscope, working at an accelerating voltage of 100 kV. TEM images were acquired by an Olympus Quemesa Camera (11 Mpx). For each sample, 5 *μ*L of freshly extracted EVs aqueous suspension was cast onto an amorphous carbon-coated Cu grid (400 mesh). Positive staining was accomplished, after the EVs deposition, by dipping the grid in a 2% (w/v) phosphotungstic acid solution for 5 seconds. Afterwards, the grid was rinsed by using ultrapure water in order to remove the excess of staining agent. The sample on the grid was left to dry over night to ensure the complete evaporation of solvent and finally stored in a vacuum chamber until TEM observation. Conversely, the negative staining was obtained, after sEVs deposition, by casting 5 *μ*L of a 2% (w/v) phosphotungstic acid solution on the grid and leaving it for 60 seconds. Staining agent excess was removed by first rinsing ultrapure water and then blotting the edge of the grid with filter paper. After completely drying the sample, the grid was kept in a vacuum chamber until TEM investigation. Size statistical analysis of the samples (sEVs average size and size distribution) was performed by means of a freeware Image J analysis program. The average sEVs size and the corresponding percentage relative standard deviation (*σ*%) were determined for each sample, to evaluate the sEVs size distribution.

### 2.6. Field Emission Scanning Electron Microscopy (FE-SEM) Investigation

The sEVs aqueous suspension was cast onto silicon chips (Ted Pella Inc.) to be processed according to the following fixation procedure, before Field Emission Scanning Electron Microscopy (FE-SEM) analysis. The sEVs were treated with 2.5% glutaraldehyde in phosphate buffer solution (PBS, 1x, pH 7.4) for 30 min and all the samples were dehydrated in 20%, 40%, 60%, 90%, and 100% ethanol in water for 5 min. Subsequently, the samples were incubated in tert-butyl alcohol and ethanol (1:1) for 5 min to complete the dehydration process. The fixed EVs were imaged by using a Zeiss Sigma FE-SEM, working at electron high tension (EHT) of 0.5-20 kV, and equipped with an “in-lens” secondary electron detector. A uniform Au metal coating of few nanometers thickness was deposited on the samples placed on silicon wafer by using a turbomolecular pumped SC7620 Mini Sputter/Glow Discharge System of Quorum Technologies, in order to limit charging artefacts and fast damage induced by electron beam of biomaterials during FE-SEM investigation.

### 2.7. Dynamic Light Scattering (DLS) and *ζ*-Potential Investigation

Evaluation of size distribution, stability, and hydrodynamic diameter of the extracted sEVs was performed by using a Zetasizer Nano ZS, Malvern Instruments Ltd., Worcestershire, UK (DTS 5.00). In particular, polydispersity index (PDI) was determined by means of DLS investigation, after sample dilution with demineralized water. Upon dilution of the sEVs samples in KCl aqueous solution (1 mM), *ζ*-potential measurements were carried out to record the surface charges of EVs samples by using a laser Doppler velocimetry (LDV). All data were reported as average values ± standard deviation, considering three replicates.

## 3. Results

### 3.1. Extracellular Vesicle Characterization

**Figure 1:**
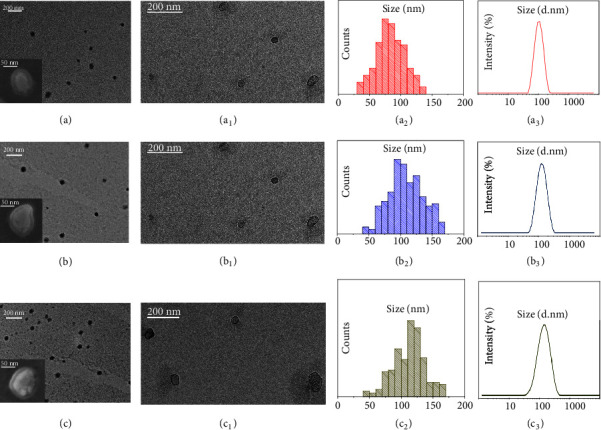
*Analysis of morphology and size distribution of freshly isolated sEVs performed by TEM, SEM, and DLS investigation*. Representative TEM micrographs obtained with positive (a, b, c) and negative (a_1_, b_1_, c_1_) staining of sEVs extracted from plasma of the healthy donors (a, a_1_) and the CRC (b, b_1_) and GC (c, c_1_) patients, along with their corresponding SEM images (Inset a, b, c). Related size distributions by TEM (a_2_, b_2_, c_2_) and DLS (a_3_, b_3_, c_3_) analysis.

sEVs were isolated by ultracentrifugation according to the protocol described in the experimental section. The freshly isolated sEVs were characterized in terms of size, size distribution, morphology, and colloidal stability by means of electron microscopies (TEM and SEM), DLS technique, and *ζ*-potential measurements (Figure 1).

Representative TEM micrographs of the positively stained EVs samples extracted from the healthy donors and the CRC and GC patients are reported in Figures 1(a), 1(b), and 1(c), respectively, showing round shaped objects that can be ascribed to the EVs and that present the same morphology regardless of the health status of the donor. The associated statistical analysis resulted in EVs average diameters of 87 nm (*σ*_%_ =22%), 108 nm (*σ*_%_ =23), and 112 nm (*σ*_%_ =21) for the healthy donors, the CRC patients, and the GC patients, respectively. Moreover, the statistical analysis highlighted a mean size of the sEVs extracted from plasma of healthy donors smaller than that of the EVs isolated from plasma of oncological patients. This trend was confirmed by DLS investigation (Figures 1(a_3_)–1(c_3_)) that resulted, for each sample, in a monomodal size distribution characterized by average hydrodynamic diameter values of 132 nm (PDI=0.125 ± 0.03), 151 nm (PDI=0.135 ± 0.035), and 157 nm (PDI=0.107 ± 0.02) for the healthy donors (Figure 1(a_3_)), the CRC patients (Figure 1(b_3_)), and the GC patients (Figure 1(c_3_)), respectively (p<0.05 average hydrodynamic diameter CRC or GC patients versus average hydrodynamic diameter of healthy control). A shift between the average size value measured from TEM and from DLS investigation can be observed for each type of EVs samples. Such a shift can be ascribed to the fact that TEM images dried sEVs, being soft organic and aqueous samples, are likely to be subjected to shrinkage as a consequence of the deposition procedure. Conversely, DLS analysis monitors the sEVs hydrodynamic diameter in solution; moreover the contribution of larger sEVs to the intensity of light scattering is more significant than the smaller ones [32].

Interestingly, TEM micrographs of sEVs samples imaged by negative staining [33–36] clearly show circular objects that can be recognized as cup-shaped membrane vesicles, which are delimited by a lipid bilayer enclosing the protein cargo, resulting from the sEVs isolation process (Figures 1(a_2_)–1(c_2_)) [37].

The same structures, reasonably accounting for spheroidal lipid bilayer-enclosed vesicles, can be also observed in the SEM images reported in the insets of Figures 1(a)–1(c) recorded for sEVs isolated from the healthy donors (inset, Figure 1(a)), the CRC (inset, Figure 1(b)), and the GC (inset, Figure 1(c)) patients, respectively.

Finally, *ζ*-potential measurements highlight the presence of negative charges onto sEVs surface, as expected for phospholipid based membrane of cells, namely, with average surface charges of (-14 ± 1) mV in the case of the healthy donors, (-17 ± 2) mV for the CRC patients, and (-16 ± 1) mV for the GC patients, in agreement with data reported in literature [38].

### 3.2. FZD-10 Expression in Extracellular Vesicles Isolated from Plasma

The levels of FZD-10 expression were investigated on samples containing the same total protein content extracted from sEVs isolated from the plasma of the 8 healthy donors. The protein samples were immunoblotted with antibodies specific for CD63, FZD-10, ALIX, and Hsp70, respectively. The Western blotting analysis confirms the presence of CD63, ALIX, and Hsp70 proteins that represent established exosomal/sEV protein markers. Indeed, housekeeping proteins, as exosomal and sEVs proteins markers, include ALIX, TSG101, the tetraspanins CD63, CD81, and CD9, HSPs, metalloproteinases, integrins, some glycoproteins, and selectins, with all of them being equivalent as a loading control for a semiquantitative analysis of immunoblotting, as confirmed in relevant reports. Here, Hsp70 was used as a loading control for the sEVs investigation. Interestingly, the blotting analysis reveals the occurrence of bands ascribable to FZD-10 in all samples, though not so evident in the healthy donor derived samples. In order to quantify the FZD-10 content in the sEVs extracted protein samples and estimate the average FZD-10 expression level in the healthy subjects, the FZD-10 bands were measured by videodensitometry analysis and normalized by using the corresponding housekeeping Hsp70 bands, for each donor [39, 40].

**Figure 2:**
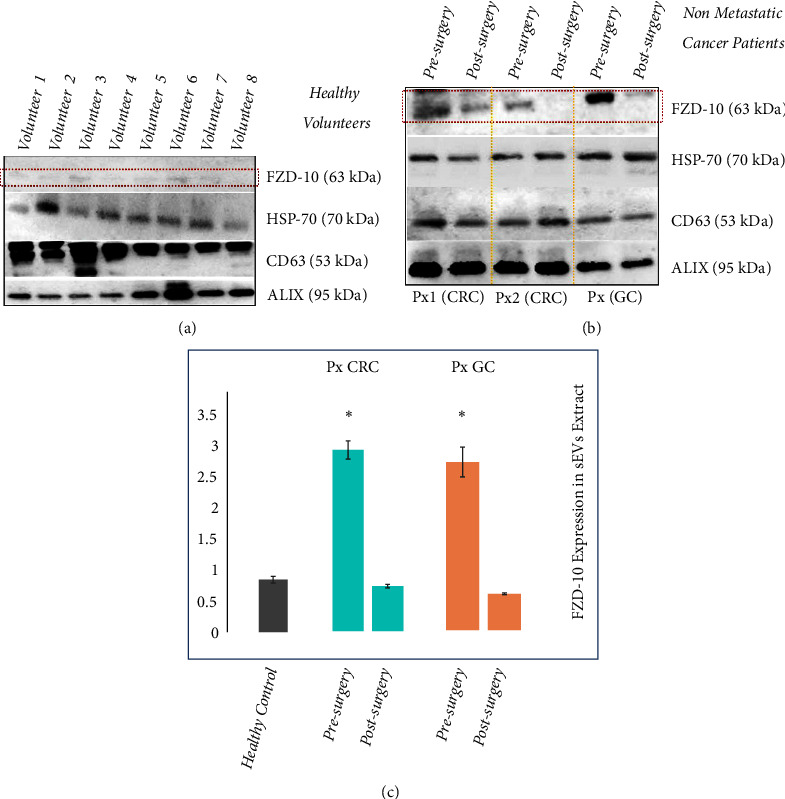
*Detection and determination of FZD-10 expression levels in sEVs isolated from healthy donors and nonmetastatic cancer patients by Western blotting and densitometry analysis*. Representative Western blotting of FZD-10 and three exosomal/EV protein markers (Hsp70, CD-63, and ALIX) in sEVs extracted from plasma of healthy donors. The same load (20 *μ*g) of samples based on total protein content (a). Western blotting of FZD-10 and three exosomal/EV protein markers (Hsp70, CD-63, and ALIX) in sEVs extracted from nonmetastatic CRC (Px1 and Px2) and nonmetastatic GC (Px3) patients, respectively. The same load (20 *μ*g) of samples based on total protein content. Molecular mass markers indicated on the right. (b). Semiquantitative evaluation of relative FZD-10 expression in sEVs extracts by densitometry analysis of protein bands in (a) and (b). FZD-10 bands were measured, upon normalization with the corresponding housekeeping Hsp70 protein band, for each patient (three replicates). Average value of FZD-10 expression levels among all patients reported in graph. (*∗*) p<0.005 versus healthy control.* n*= 16 for CRC,* n*=2 for GC, and* n*=8 for healthy donors (c).

Such preliminary semiquantitative analysis resulted in an average value of 0.784 ± 0.05 ratio that determines the maximum value for the FZD-10 content that can be considered normal in healthy subjects (Figure 2(a)) [41]. The healthy control value, obtained as average value of FZD-10 expression level in the 8 healthy donors, was reported in Figures 2(c), 2(b), and 4(b).

The protein sEVs samples from plasma of 16 nonmetastatic CRC patients and 2 nonmetastatic GC patients that were considered free from the disease after surgery, were investigated for the detection and determination of FZD-10, along with the exosomal/sEV protein markers.

In particular, Western blotting analysis was carried out on the proteins extracted from the sEVs from plasma of the patients, collected 24 hours before and 30 days after the surgical resection, during disease revaluation (Figure 2(b)). The semiquantitative investigation, performed by means of videodensitometry, resulted in an average level of FZD-10 expression of 2.648 ± 0.134 ratio before the surgery and of 0.658 ± 0.024 ratio 30 days after the intervention, for the patients affected by CRC, and 2.413 ± 0.213 ratio before the surgery and of 0.520 ± 0.032 ratio after 30 days the intervention, for the patients affected by GC, all compared to the healthy control 0.784 ± 0.05 (Figure 2(c)).

An increase of the expression level of FZD-10 is seen in the oncological patients when compared to the level of the protein for the healthy control before surgery, while a decrease of the FZD-10 relative content was detected for the same patients after the intervention.

**Figure 3:**
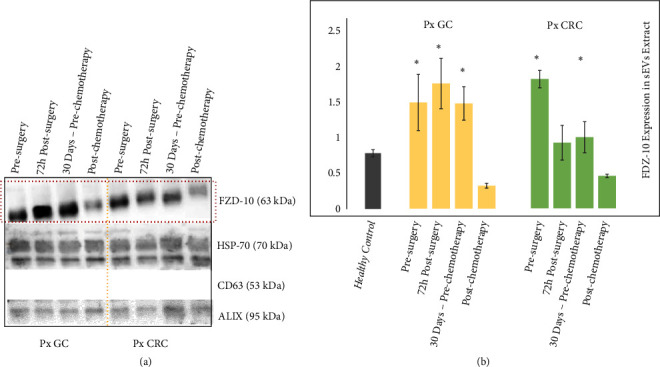
*Detection and determination of FZD-10 expression levels in sEVs isolated from cancer patients with metastasis by Western blotting and densitometry analysis*. Representative Western blotting of FZD-10 and three exosomal/EV protein markers (Hsp70, CD-63, and ALIX) in sEVs extracted from patients with metastatic GC (Px GC) and metastatic CRC (Px CRC), before surgery and at different treatment steps. Molecular mass markers indicated on the right. The same load (20 *μ*g) of samples based on total protein content (a). Semiquantitative evaluation of relative FZD-10 expression in sEVs extracts by densitometry analysis of protein bands in (a). FZD-10 bands were measured and normalized with corresponding housekeeping Hsp70 bands, for each CRC and GC patient (three replicates). Average value of FZD-10 expression levels among all CRC or GC or metastastic CRC patients reported in graph. (*∗*) p<0.005 versus healthy control.* n*= 6 for CRC,* n*=6 for GC, and* n*=8 for healthy donors (b).

The protein sEVs samples from 6 metastatic CRC patients and 6 metastatic GC patients, at different phases of medical treatment, were investigated for the detection and quantitation of FZD-10, along with the exosomal/EV protein markers (Figure 3).

In particular, sEVs samples from each patient were obtained 24 h before surgery, 72 h after surgery, and 30 days after surgery, namely, before chemotherapy cycle for the GC patients and before the metastasis removal for the subjects affected by CRC. Finally, sEVs samples were attained for each patient 30 days after the end of the treatment, either chemotherapeutic or surgical, when they were considered free from disease.

The Western blotting analysis resulted in a level of expression of FZD-10 in the EVs from the GC patients of 1.49 ± 0.396 ratio before surgery, 1.765 ± 0.356 ratio 72 h after surgery, 1.484 ± 0.236 ratio 30 days after surgery, before chemotherapy, and finally 0.324 ± 0.034 30 days after the end of the treatment.

The investigation performed on the sEVs from the CRC patients determined an FZD-10 expression level of 1.775 ± 0.120 ratio before surgery, 0.900 ± 0.239 72 h after surgery, 0.976 ± 0.213 ratio after 30 days before metastasis removal, and finally 0.444 ± 0.023 ratio after the end of the treatment.

For all patients an average level of FZD-10 expression higher than the value for the healthy control was observed. Interestingly, for the GC patients the value measured 72 hours after the surgery appears to further increase with respect to the level before the intervention, remaining, however, still higher than the healthy control value. The FZD-10 expression for the GC patients was then found to decrease 30 days after the surgery, before the chemotherapy, to, finally, turn to a level comparable to the average value measured for the healthy control at the end of the treatment.

A different trend was observed for the level of FZD-10 protein expression in the samples from the CRC patients: 72 hours after the surgery the value decreased compared to the level recorded before the intervention, then kept decreasing considering the value at 30 days after the intervention, before the metastasis removal, which however was still higher than the control, and finally at the end of the treatment (Figures 3(a) and 3(b)) reached a level comparable to the average value measured for the healthy control.

### 3.3. Comparison between FZD-10 Expression Level in Small Extracellular Vesicles and in Whole Plasma

**Figure 4:**
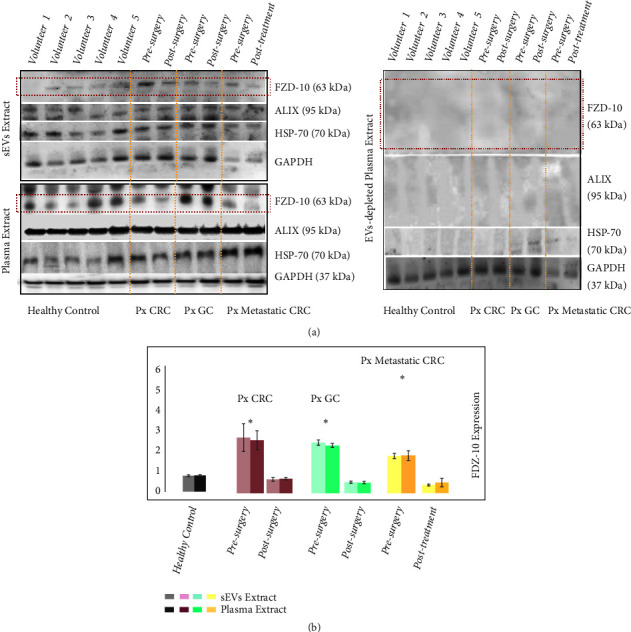
*Detection and determination of FZD-10 expression levels in sEVs and in whole plasma samples of healthy donors and cancer patients by Western blotting and densitometry analysis*. Representative Western blotting of FZD-10, two exosomal/EV protein markers (Hsp70 and ALIX) and GADH in sEVs, whole plasma and EVs-depleted plasma of healthy donors, CRC patient (before and after surgery), and GC patient (before surgery and after pathology resolution). Molecular mass markers indicated on the right. The same load (20 *μ*g) of samples based on total protein content (a). Semiquantitative evaluation of relative FZD-10 expression in sEVs extracts and whole plasma by densitometry analysis of protein bands in (a). FZD-10 bands were measured, upon normalization with corresponding housekeeping Hsp70 and GAPDH bands in the case of sEVs and plasma samples, respectively, for each CRC, GC, and metastatic CRC patient (three replicates). Average value of FZD-10 expression levels among each group of subjects reported in graph. (*∗*) p<0.005 versus healthy control.* n*= 16 for CRC,* n*=2for GC,* n*=6 for metastatic CRC, and* n*=8 for healthy donors (b).

Finally, the Western blotting investigation was also carried out on whole plasma protein samples from each patient and from the healthy donors, in order to detect and evaluate the level of FZD-10 protein therein and thus allow a comparison with the FZD-10 protein value determined in the sEVs samples from the same donors (Figure 4).

The evaluation of the FZD-10 protein level of expression was performed considering the whole plasma proteins and the EVs samples of, respectively, 8 healthy donors and 16 nonmetastatic CRC, 2 nonmetastatic GC, and 6 metastatic CRC patients. In Figure 4(a), representative Western blotting of FZD-10, Hsp70, ALIX, and GADH in EVs and whole plasma of healthy donors and oncological patients, before surgery and after pathology resolution, is reported in the top and bottom of the left panel, respectively. Investigation on the content of the same proteins was performed by Western blot analysis in the EVs-depleted plasma (Figure 4(a), right panel). In Figure 4(b), histograms obtained by semiquantitative evaluation of relative FZD-10 expression in sEVs extracts and whole plasma by densitometry analysis of protein bands in Figure 4(a) (left panel) are shown. While the FZD-10 protein relative content in sEVs extracts was determined by normalizing the result of the Western blotting with respect to the Hsp70, in the case of whole protein plasma specimens the FZD-10 protein relative content was evaluated by normalizing the result of the Western blotting with respect to GAPDH protein, purposely selected as it is a housekeeping protein present both in plasma and in the sEVs [42]. The maximum value for the FZD-10 content estimated by semiquantitative analysis of the whole protein plasma specimens of healthy subjects resulted in an average value of 0.798 ± 0.04 ratio, which was comparable to the average value achieved in the sEV protein extract (0.784 ± 0.05 ratio). FZD-10 expression level determined by investigation performed on the sEV and the whole plasma of nonmetastatic CRC patients resulted in 2.648 ± 0.134 ratio and 2.508 ± 0.45 ratio, respectively, before the surgery, and finally 0.658 ± 0.024 ratio and 0.692 ± 0.054 ratio, respectively, after surgery. The semiquantitative analysis carried out on the sEV and the whole plasma specimens of metastatic CRC patients resulted in a FZD-10 expression level of 1.775 ± 0.120 ratio and 1.869 ± 0.210, respectively, before surgery, and 0.444 ± 0.023 ratio and 0.581 ±.156, respectively, after pathology resolution (Figure 4(a), left panel, and Figure 4(b)). In the case of nonmetastatic GC patients, FZD-10 content was estimated to be, before surgery, equal to 2.413 ± 0.213 ratio and 2.122 ± 0.109 ratio in sEV and whole plasma extracts, respectively, and, after surgery, 0.520 ± 0.032 ratio and 0.504 ± 0.058 ratio.

In all the investigated cases, the values of FZD-10 expression in the whole plasma and in the sEVs samples for the same subject were found comparable, irrespectively of the health status (Figure 4(a)). FZD-10 expression level was found not detectable in the sEV depleted plasma extracts neither from the healthy donors nor from the oncological patients.

## 4. Discussion

EVs have been shown to play a clear role as active messengers and mediators of intracellular communication during tumor progression and spreading, being involved in mechanism such as tumor microenvironment maturation, metastatic dissemination, and anticancer therapies resistance [43].

Here, the occurrence of Frizzled-10 protein in EVs from patients affected by different type of sporadic cancer at different stages of disease, before and after treatments, with different etiology and progression, was examined and investigated.

The FZD proteins have been found to play an important role in the Wnt transduction signal, during embryonic development, in stem cell homeostasis both in normal and injured tissues, also in the case of cancer diseases [44–46]. The Wnt cascade and consequently also the Frizzled receptor complex were involved in the *α*/*β*-catenin control which connects cadherins to the actin cytoskeleton, control junction stability, and finally variation of cell plasticity during the carcinogenesis [47].

A different expression of the FZD-10 level in tissues from patients affected by different oncological diseases, including CRC and GC, has been recently reported [23].

However, only little evidence has been found on the putative mechanism for signal transmission mediated from FZD-10 in cancer pathology. In fact, the indications on the role of FZD proteins were only limited to their involvement in an autocrine mechanism detected in the cancer tissue and microenvironment component [48].

In this work, for the first time, FZD-10 protein was found to be specifically localized in the sEVs from plasma of both oncological and healthy subjects, and not just in the canonical localization, such as cell membrane and/or cytoplasm, as reported by Scavo et al. [23]. Moreover, interestingly, the level of the protein expression in oncological patients was found higher than that recorded for the healthy control group.

The experiments were carried out considering sEVs from patients affected by CRC and GC, both metastatic and nonmetastatic, before and after the surgery, at the end of the treatment, either chemotherapy or metastasis surgical removal, and at the disease resolution, as well as from healthy donors.

From this perspective, it was considered relevant to elucidate the properties of the sEVs from plasma of both healthy and oncological subjects in terms of morphology, surface charge, average size, and size distribution of sEVs, as these characteristics may result in distinctive features, useful not only for assessing the successful sEVs purification process, but also for possible future diagnostic purposes.

The average size value of the EVs, derived from the statistical analysis achieved either from TEM or DLS investigation, was compatible with those found in literature reports, obtained by using the same techniques, respectively, as ascribable to the smallest EVs population, represented by exosomes [49]. Moreover, the characteristic cup-shaped morphology of the extracted sEVs, highlighted by TEM and SEM analysis, accounts for spheroidal lipid bilayer-enclosed vesicles, which have been reported as distinctive features typically ascribed to exosomes.

It is also worth noting that both TEM and DLS investigation highlighted a mean size for sEVs from healthy donors smaller than that detected for those isolated from oncological patients. The obtained results suggest a possible correlation between the average size of the sEVs and the health condition of the donor. Such a hypothesis, although supported by a recent report that proved a significant difference in urinary exosome sizes between healthy controls and patients with prostate cancer by means of the flow field-flow fractionation technique [50], would need to be further investigated.

Interestingly, the overall results obtained by Western blotting analysis performed on sEVs isolated from both the healthy donors and the CRC or GC patients indicated that the FZD-10 level of expression in oncological patients was higher than that recorded for the healthy control group, while the protein level in the sEVs from patients at the end of the treatment appears to have values comparable with the average level recorded for the healthy donors. Therefore, the FZD-10 level of expression represents a clear indication of the pathological condition.

In particular, for the nonmetastatic oncological, either GC and CRC, patients, the FZD-10 in EVs reached already after the surgery a level compatible with the value recorded for the healthy control. For the metastatic oncological patients the investigation was carried out throughout the treatment, also after the first not resolving intervention, in order to monitor the level of the FZD-10 protein in the EVs at the different phases.

For the metastatic CRC oncological cases, the protein level appeared to decrease already immediately after the surgery, to keep then decreasing throughout the subsequent metastasis removal phase, and to get down to the normal level at the very end of the treatment.

Remarkably, in the case of EVs samples from metastatic GC patients, after 72 hours from surgery an increase of the FZD-10 level was observed, instead. Such evidence can be explained assuming that FZD-10 may behave similarly to other cancer markers that tend to increase their level of expression after intervention, as a consequence of a postsurgical inflammatory state [10, 51]. Such an explanation is supported by the evidence that 30 days after the surgery, already before the chemotherapy, the FZD-10 expression level decreased, although not to the normal value, which is, in fact, reached only at the very end of the treatment, that is, 30 days after the chemotherapy.

For each subject, either oncological patients or healthy donors, the level of FZD-10 found in the specimens of the total proteins extracted from the whole plasma was comparable with that of the protein in EVs from the same subject, thus clearly indicating that FZD-10 in the plasma is uniquely carried by EVs, rather than present in the whole plasma. In particular, the specimens of the proteins extracted from the whole plasma samples presented a level of FZD-10 expression higher for oncological patients than for the healthy control group, to finally stabilize within the normal value range at the end of the treatment. This result demonstrated that FZD-10 detected in the EVs represents the total actual protein level in the plasma; therefore the determination of the FZD-10 level of expression could be directly performed on the whole plasma samples, without any laborious EVs extraction procedures.

Based on the obtained results and on the evidence reported in the relevant literature, the enrollment of FZD-10 containing EVs on the control of cancer evolution and cancer cell modification can be confidently established. Therefore, the EVs delivered FZD-10 can be indicated as a new and valid prognostic marker and represent a potential valuable tool to accomplish early diagnosis of cancer and to monitor the efficacy of its treatment and management. Indeed, a simple, fast, and noninvasive diagnostic test could be developed starting from plasma, an easily available biological fluid. In this perspective further investigation could be performed to monitor cell morphological variations, to assess the paracrine control of the Wnt cascade mediated by extravesicular FZD-10, on the remodeling of tumor cells.

Future study will be aimed at extending the cohort of patient, validating the FZD-10 protein as a molecular marker for monitoring status of patients at different treatment stages (i.e., presurgery state and postsurgery treatment cycle of chemotherapy, or radiotherapy and canonical follow-up), and evaluating the possibility of applying FZD-10 as a marker for other different types of cancer such as cholangiocarcinoma, hepatocellular carcinoma, and pancreatic carcinoma.

## Figures and Tables

**Table 1:** Patients index with age, sex, diagnosis, and marker.

COLORECTAL CANCER	AGE	SEX	DIAGNOSIS	STAGE TNM	STAGE AJC	GRADING	LIVER METASTASIS
1	71	m	CRC	pT1N0M0	I	G1	N
2	90	f	CRC	pT1N0M0	I	G1	N
3	59	m	CRC	pT2N0M0	I	G1	N
4	90	f	CRC	pT2N0M0	I	G2	N
5	71	f	CRC	pT2N0M0	I	G2	N
6	73	m	CRC	pT2N0M0	I	G2	N
7	49	m	CRC	pT2N1bM0	IIIA	G3	N
8	68	f	CRC	pT2N1bM0	IIA	G2	N
9	78	f	CRC	pT2N0M0	I	G2	N
10	72	m	CRC	pT3N0M0	IIA	G2	N
11	63	m	CRC	pT3N2aM1	IVA	G3	Y
12	77	m	CRC	pT3N0M0	IIA	G2	N
13	82	m	CRC	pT3N1bM0	IIIB	G2	N
14	72	m	CRC	pT3N2aM1	IVA	G2	Y
15	66	m	CRC	pT3N0M0	IIA	G2	N
16	70	f	CRC	pT3N0M1	IIA	G2	N
17	59	m	CRC	pT4aN2aM1	IVA	G2	Y
18	72	m	CRC	pT4aN2aM1	IVA	G2	Y
19	71	m	CRC	pT4aN2bM1	IVA	G2	Y
20	79	f	CRC	pT4bN2bM0	IIIC	G2	N
21	69	m	CRC	pT4aN1aM1	IVA	G2	Y
22	67	f	CRC	pT4bN1bM0	IIIB	G2	N

GASTRIC CANCER							
1	72	m	GC	pT4aN0M0	IIB	G2	N
2	73	m	GC	pT4bcN3M1	IV	G3	Y
3	76	m	GC	pT4aN1M1	IV	G2	Y
4	65	f	GC	pT3N0M0	II	G2	N
5	67	f	GC	pT4aN1aM1	IV	G2	Y
6	54	m	GC	pT4aN2aM1	IV	G2	Y
7	63	m	GC	pT4aN1M1	IV	G2	Y
8	71	f	GC	pT4aN1bM1	IV	G2	Y

## Data Availability

All data used to support the findings of this study are included within the article.

## Abbreviations

CRC: Colorectal cancer
GC: Gastric Cancer
EVs: Extracellular vesicles
FZD: Frizzled proteins
EDTA: Ethylenediaminetetraacetic acid
UC: Ultracentrifugation
DLS: Dynamic light scattering
FE-SEM: Field emission scanning electron microscopy
EHT: Electron high tension
TEM: Transmission electronic microscopy
RIPA: Radio immunoprecipitation buffer.
