# Erratum to “Frizzled-10 Extracellular Vesicles Plasma Concentration Is Associated with Tumoral Progression in Patients with Colorectal and Gastric Cancer”

**DOI:** 10.1155/2020/6153432

**Published:** 2020-01-30

**Authors:** Maria Principia Scavo, Antonio Cigliano, Nicoletta Depalo, Elisabetta Fanizza, Maria Grazia Bianco, Nunzio Denora, Valentino Laquintana, Maria Lucia Curri, Dionigi Lorusso, Claudio Lotesoriere, Alba Panarese, Gianluigi Giannelli

**Affiliations:** ^1^Personalized Medicine Laboratory, National Institute of Gastroenterology “S. De Bellis” Research Hospital, Via Turi 27, Castellana Grotte, Bari, Italy; ^2^Institute for Chemical-Physical Processes (IPCF)-CNR, SS Bari, Via Orabona 4, Bari 70125, Italy; ^3^Università Degli Studi di Bari Aldo Moro, Dipartimento di Chimica, Via Orabona 4, Bari 70125, Italy; ^4^Università Degli Studi di Bari Aldo Moro, Dipartimento di Farmacia, Scienze Del Farmaco, Via Orabona 4, Bari 70125, Italy; ^5^Surgical Gastroenterology Unit, National Institute of Gastroenterology “S. De Bellis” Research Hospital, Via Turi 27, Castellana Grotte, Bari, Italy; ^6^Oncology Unit, National Institute of Gastroenterology “S. De Bellis” Research Hospital, Via Turi 27, Castellana Grotte, Bari, Italy; ^7^Endoscopy Unit, National Institute of Gastroenterology “S. De Bellis” Research Hospital, Via Turi 27, Castellana Grotte, Bari, Italy; ^8^Scientific Direction, National Institute of Gastroenterology “S. De Bellis” Research Hospital, Via Turi 27, Castellana Grotte, Bari, Italy

In the article titled “Frizzled-10 Extracellular Vesicles Plasma Concentration Is Associated with Tumoral Progression in Patients with Colorectal and Gastric Cancer” [1], there was an error in Figures 1 and 2. Because of an error in figure assembly after acceptance, in Figure 1, the two images (a1) and (b1) were identical, and in Figure 2, the expression of the CD63 was missing.

The correct figures should be as follows:

## Figures and Tables

**Figure 1:**
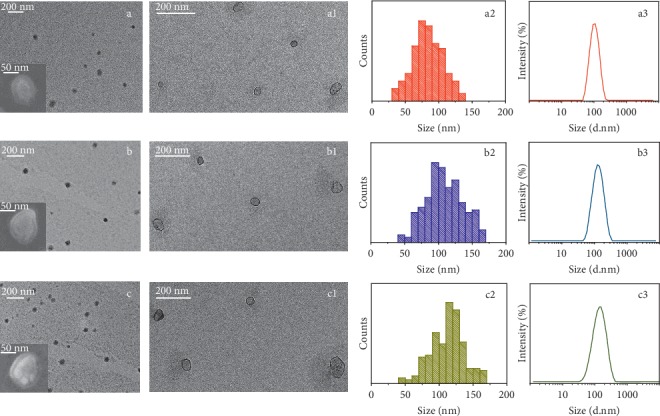
Analysis of morphology and size distribution of freshly isolated sEVs performed by TEM, SEM, and DLS investigation. Representative TEM micrographs obtained with positive (a, b, c) and negative (a1, b1, c1) staining of sEVs extracted from plasma of the healthy donors (a, a1) and the CRC (b, b1) and GC (c, c1) patients, along with their corresponding SEM images (inset a, b, c). Related size distributions by TEM (a2, b2, c2) and DLS (a3, b3, c3) analysis.

**Figure 2:**
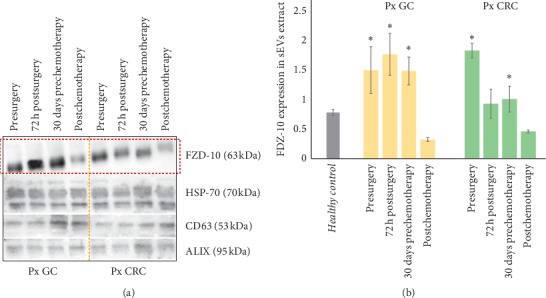
Detection and determination of FZD-10 expression levels in sEVs isolated from cancer patients with metastasis by Western blotting and densitometry analysis. Representative Western blotting of FZD-10 and three exosomal/EV protein markers (Hsp70, CD-63, and ALIX) in sEVs extracted from patients with metastatic GC (Px GC) and metastatic CRC (Px CRC), before surgery and at different treatment steps. Molecular mass markers are indicated on the right. The same load (20 *μ*g) of samples based on total protein content (a). Semiquantitative evaluation of relative FZD-10 expression in sEVs extracts by densitometry analysis of protein bands in (a). FZD-10 bands were measured and normalized with corresponding housekeeping Hsp70 bands, for each CRC and GC patient (three replicates). Average value of FZD-10 expression levels among all CRC or GC or metastastic CRC patients reported in graph. *p* < 0.005 versus healthy control. *n* = 6 for CRC, *n* = 6 for GC, and *n* = 8 for healthy donors (b).

## References

[1] Scavo M. P., Cigliano A., Depalo N. (2019). Frizzled-10 extracellular vesicles plasma concentration is associated with tumoral progression in patients with colorectal and gastric cancer. *Journal of Oncology*.

